# Would You Trust Driverless Service? Formation of Pedestrian’s Trust and Attitude Using Non-Verbal Social Cues

**DOI:** 10.3390/s22072809

**Published:** 2022-04-06

**Authors:** Suji Choi, Soyeon Kim, Mingi Kwak, Jaewan Park, Subin Park, Dongjoon Kwak, Hyun Woo Lee, Sangwon Lee

**Affiliations:** 1 Department of Human Environment and Design/Human Life Innovation Design, Yonsei University, Seoul 03722, Korea; charcalling@yonsei.ac.kr (S.C.); skim242@yonsei.ac.kr (S.K.); rhkr01@yonsei.ac.kr (M.K.); 2 Department of Design Science, Graduate School of Techno Design, Kookmin University, Seoul 02707, Korea; jaewanpark@kookmin.ac.kr; 3 Department of Human Environment and Design, Yonsei University, Seoul 03722, Korea; psg200999@yonsei.ac.kr (S.P.); dongjoonkwak@gmail.com (D.K.); 4 Department of Construction Management, University of Washington, Seattle, WA 351610, USA; hyunwlee@uw.edu

**Keywords:** autonomous vehicles, driverless service, pedestrian–AV interaction, social cues, trust, intimacy, brand attitude

## Abstract

Despite the widespread application of Autonomous Vehicles (AV) to various services, there has been relatively little research carried out on pedestrian–AV interaction and trust within the context of service provided by AV. This study explores the communication design strategy promoting a pedestrian’s trust and positive attitude to driverless services within the context of pedestrian–AV interaction using non-verbal social cues. An empirical study was conducted with an experimental VR environment to measure participants’ intimacy, trust, and brand attitude toward AV. Further understanding of their social interaction experiences was explored through semi-structured interviews. As a result of the study, the interaction effect of social cues was found, and it was revealed that brand attitude was formed by the direct effects of intimacy and trust as well as the indirect effects of intimacy through trust’s mediation. Furthermore, ‘Conceptual Definition of Space’ was identified to generate differences in the interplay among intimacy, trust, and brand attitude according to social cues. Quantitative and qualitative results were synthesized to discuss implications considering the service context. Practical implications were also addressed suggesting specific design strategies for utilizing the sociality of AV.

## 1. Introduction

With the development of Autonomous Vehicle (AV) technology, research on AV including human factors [1,2,3,4,5] has been actively conducted. The scope of research on AV has expanded to various interactions regarding complex road environments [6]. Early research on AV focused on safe driving and efficient interaction between the driver and the AV [7,8,9,10,11,12]. Recent research examined more diverse interactions not only covering in-vehicle but also with-vehicle interactions [3]. Such research includes the study on factors that support the passenger’s non-driving activities [8] or the study examining other users of the transportation network [13], including pedestrians or other cars.

The problem of noticeable skepticism in society [2] and human trust towards AV remains despite the wide range of applications of AV in transportation services. Using self-driving technology and artificial intelligence applied to AV, various services, such as shared taxis, autonomous public buses, and delivery services, have emerged. As AV keeps spreading widely, there arises a need to induce people’s trust in AV leading to positive perception and acceptance of it [14]. Compared to direct users, pedestrians have a relatively lower understanding of the service and the technological ability of AV. Thus, it is required to develop a communication strategy that allows the formation of trust and a positive attitude in a momentary interaction. 

There has been relatively little research carried out on pedestrian–AV interaction and trust within the context of service provided with AV. Most of the research on trust within the context of AV dealt with drivers [2,14,15] or the delivery and acceptance of safety information [1,5]. A few studies expanded the scope of trust research by providing a general conceptual framework encompassing the overall landscape of self-driving [16]. However, empirical studies examining the effect of design variables during the interaction with pedestrians deserve scholarly attention still.

At the outset, it is necessary to clarify the study population for the pedestrian group. In general terms, age is a crucial factor in the study of human trust toward AV [8]. According to Charness’s research team, younger adults (18–34) tend to concern less about AV technology [17]. Furthermore, younger adults prefer sharing mobility, public transportation, and AV technology whereas the older generation (i.e., generation X) relies more on private vehicles [18]. This study pays attention to how the group with a generally positive attitude toward AV technology will respond according to detailed design elements.

This study explores the communication design strategy that allows a pedestrian’s formation of trust and positive attitude towards the service AV provides within a context of pedestrian–AV interaction. Assuming interaction between pedestrians and AV to be brief and spontaneous, the current study focuses on the effects of non-verbal social cues. Social interaction and the formation of intimacy are also explored to discuss the pedestrian’s subjective attitude. In turn, the proposed research questions are as follows.

RQ1. How do the non-verbal social cues affect a pedestrian’s trust and subjective/emotional attitude toward the service provided by AV during the social interaction?

RQ2. How does the relationship between a pedestrian’s trust and subjective/emotional attitude vary according to the non-verbal social cues?

RQ3. What are the associated constructs that affect a pedestrian’s subjective/emotional attitude and how do they relate mutually?

## 2. Background

### 2.1. A Review of Previous Studies

#### 2.1.1. Trust Formation in Social Pedestrian-AV Interaction

Trust is a construct based on a relationship [8,19]. The construct was used to address diverse relationships as the research on trust expanded and linked to other fields. As for what trust is, multiple definitions have been provided by previous research. Lee and See have organized the different definitions into four categories: belief, attitude, intention, and behavior [20]. A framework that encompasses said definitions has been developed by Ajzen and Fishbein [21]. It demonstrates that belief leads to attitude, which then serves as a basis for intention, resulting in action. Belief is a common theme in prior studies as it is the first step in the formation of trust. Thus, the following definition of trust is chosen for this study: trust is the expectation of technically competent role performance [22]. Interpersonal trust [8] was applied to system management and was studied as organizational trust dealing with intra-organizational or inter-organizational relations [19,23]. It was also expanded into the field of strategic communications and public relations studies [24]. Trust of non-human beings, i.e., human–computer trust (HCT), was also introduced and explored [24] with the growing importance of interaction between human and computer systems. Recently, with the wide use of related services and agents both equipped with artificial intelligence-based decision-making support systems, studies on the formation of trust, and relationships between people and agents are emerging [25,26].

With the growth of AV technology, the formation of trust between humans and AV has gained the interest of contemporary researchers. Most of the studies on trust in the context of a self-driving focus on interaction with drivers or conveyance and acceptance of safety information: driver-focused research to effectively apply self-driving technology to the transportation industry [2,15], research investigating the driver’s experience [14], and research focusing on the interface as a part of human–machine interaction research [1]. Raats et al. expanded the scope of trust research by providing a general conceptual framework that encompasses the overall landscape of self-driving [4]. However, empirical studies on the effect of design variables within a specific context of pedestrian–AV interaction still require more attention. A pedestrian–AV interaction study conducted by She et al. explored the influence of information manipulating AV’s communication style, speed information, and adaptive communication strategy on trust in the decision-making process of pedestrians [5]. However, considering the aim of this study to explore strategies to build instantaneous trust, positive image, and attitude toward services rather than to aid, the prior has the limitation of focusing on the delivery of auditory or simple visual information (e.g., text).

The concept of trust between AV and pedestrian needs to be addressed within the context of a relatively short period of interaction. Trust based on relationships is generally defined as positive expectations for objects and a willingness to take risks [19,27]. However, it is necessary to take a more multidimensional perspective on defining the construct regarding the subject of interaction and context [23]. The study on organizational trust within a relatively large range of organizational environments deals with long-term trust formation [19]. Within the context of human–computer interaction (HCI), Madsen and Gregor conceptualized and categorized relationship types (Macro and Micro) and trust types (Cognition Based and Affect Based) [7]. Cognition-based trust is a subjective impression formed instantaneously within a relatively short period of time [18]. Such a construct is based on the other’s ability, reliability, knowledge, and competence [28]. On the other hand, affect-based trust relates to emotional bonds [23], which develops when sufficient information about the other is delivered and effective communication is achieved [24,27]. 

This study will define trust as a cognition-based trust considering the momentary nature of the interaction between AV and pedestrians and the tendency of pedestrians to perceive trust with a subjective impression of AV. Adopting the definition of trust provided in said research and the general concept of trust defined above, HCT covered in this study is defined to be the extent to which a user is confident in the recommendations, actions, and decisions of an artificially intelligent decision aid, dealing with the cognitive/belief aspect of trust.

In the context of HCT, social interaction between humans and agents is an effective approach to the formation of trust [29]. This suggests the need for a study on social pedestrian–AV interaction defining AV as an entity with sociality. Previous studies on interactions with sociable robots and trust formation [30,31,32] focused on the experiences of information delivery or acceptance leaving room for studies on the application of sociality. While a few studies on AV’s social behavior exist [10], research on AV with sociality or their formation of a relationship with humans still needs further exploration.

#### 2.1.2. Non-Verbal Social Cues for the Formation of Trust through Intimacy

An agent’s social behavior affects the relationship development between humans and agents [29]. Specifically, an agent’s social behavior induces the interactor’s formation of intimacy and trust resulting in an acceptance of the technology. Intimacy, defined as the closeness and familiarity recognized during the interaction, [33], operates as the preceding factor for forming trust on entities with sociality [34]. Along with trust, intimacy has been identified as a significant factor in forming a positive attitude toward a service [35]. Thus, the effect of the sociality of AV on trust and intimacy needs to be explored, for the impression formed in a moment (i.e., positive emotion, attitude) is important.

The sociality of agents, computers, systems, and robots has been explored in the context of non-verbal signals [29,36,37]. Patterson divided non-verbal components into static and dynamic [38]. The former includes settings and appearance, while the latter includes distance/orientation, gaze, facial expressions, posture/movement, touch, vocal characteristics, and olfactory cues. In a similar context, Feine et al. explained the non-verbal signals and sociality of conversational agents utilizing the concept of social cues [39]. Social cues are classified into four categories (i.e., verbal, visual, audio, and invisible). Visual social cues consistent with the scope of this study include kinesics (movement of agent’s body parts), proxemics (background and conversational distance), agent appearance (graphical representation), and computer-mediated communication (emoticons and typefaces).

Among many non-verbal social cues, ‘dynamic proxemics’ is a crucial design component to be addressed in our study. Spatial characteristics and proxemics are important properties for AV that inevitably encounters pedestrians while driving on the road. In particular, the distance between pedestrians and AV is an important factor for the pedestrian to feel momentarily safe and form trust [40]. There are few studies, however, dealing with the concept of conversational distance in the context of AV actively trying to establish a social relation with pedestrians as well as studies exploring the interaction effects of other non-verbal signals.

Eye movement, a sub-element of kinesics suggested by Feine et al. [39], has been examined by several scholars in human–robot interaction (HRI) highlighting the importance of the interaction effect between proxemics (i.e., the physical distance between human and robot) and gaze [41,42,43,44,45]. In addition, AV’s eyes are the design element that can effectively interact with pedestrians through minimal movement in situations where there is no other part of the AV’s body that can move freely.

#### 2.1.3. Brand Attitude as a Subjective/Emotional Attitude toward Service

With the development of artificial intelligence and its vast commercialized applications (e.g., AV, voice assistant, image search engines, curated shopping), studies have been conducted on the relationship between its application and the customer’s service experience [46]. Service experience is related to the subjective and emotional response and attitude of anyone who encounters information related to the brand, which is the provider of service beyond the direct customer and user of the service [47,48]. In this study, brand experience in the context of service is defined as a concept that deals with comprehensive responses and attitudes toward services provided by AV.

There have been numerous concepts to explain one’s response towards products or services provided by brands. Brand awareness is an individual’s ability to recall or notice a particular brand among various information during the moment of purchase [49,50]. Thus, the concept assumes one’s repetitive exposure to brands providing similar services or products. While brand awareness is related to the cognitive evaluation of a brand, brand image is an overall evaluation one makes of a particular brand. It is the comprehensive and cumulative impression formed by one’s beliefs on attributes, benefits from a brand, and attitude towards it [51]. Brand attitude is defined as the disposition towards a brand that leads to a particular manner of behavior consistently displayed [52,53]. Compared to the prior concepts introduced (i.e., brand awareness, brand image), brand attitude is effective to capture one’s evaluation towards a brand providing newly introduced services or products that people lack everyday experiences. Therefore, this study utilizes brand attitude as it is effective to measure one’s response towards a service in our study context. 

### 2.2. Research Model

**Figure 1:**
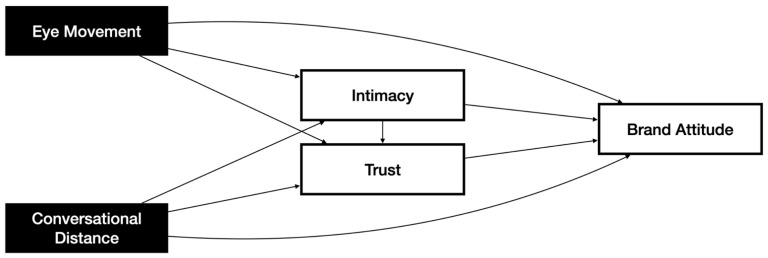
Research Model.

The research model derived through the review of previous studies is shown in Figure 1. Trust is defined as a subjective impression of the reliability and competence of objects recognized through interaction within a relatively short time [24]. Intimacy is defined as a variable meaning emotional proximity recognized through interaction with an object in a relatively short time [29,33], and preceding trust [34]. Trust mediates the influence of intimacy on brand attitude. According to Hwang and Hyun [1], brand attitude is the concept representing a temporary subjective/emotional attitude toward a service brand formed through interaction with an AV, the physical service provider. 

In this study, non-verbal social cues refer to conversational distance and eye movement. According to Feine et al. [39], conversational distance is defined as follows: the physical distance between a static AV and a pedestrian. Related to the distance, the space around a person is categorized into four types: intimated distance (~0.45 m); personal distance (0.45~1.2 m); social distance (1.2~3.5 m); public distance (3.5 m~) [54]. In our study, the conversational distance was divided and operated into two categories: near (1 m) and far (3.5 m). Near conversational distance refers to the vehicle actively approaching within the personal distance of the pedestrian for conversation, while far refers to the vehicle carefully keeping its distance within the boundary of social zones for conversation. Meanwhile, following previous studies [42,43,44,45], eye movement in our study means eyes blinking and gazing. This variable is classified as yes or no. Yes indicates the cases when the vehicle moves its eyes to make a contact while no indicates when the vehicle has static eyes without any recognizable social signals.

## 3. Methods

### 3.1. Stimuli

#### 3.1.1. Design and Development

Among the various services utilizing AV, the latest cases were investigated to select the service area for this study. Behance, a platform for designers to upload creative concept design results, was used to select cases that are likely to be commercialized soon. Among the results when searching for ‘Autonomous Vehicle Service’ as keywords on Behance, the top 30 were collected based on ‘Most Discussed’. This criterion was used to preferentially collect cases gaining attention from the related field. The date and time of the search were 00:00 (KST) on 1 September 2021. Among the top 30 results, three researchers conducted filtering and categorizing to extract and classify cases of services using AV. In total, 12 cases of simple exterior sketches and 6 cases of UI/UX design of private cars were excluded. The remaining 12 categories were classified into delivery service and public transportation service.

These two categories (i.e., delivery service, public transportation service) were selected for specific scenarios of the experiment. Delivery service and public transport, in particular, are the areas where companies have constantly invested in BOSH [55]; Navya [56]; Domino [57]; Walmart [58]; Uber [59]. In fact, the future of delivery by an autonomous car and heading to work on a driverless shuttle is coming close. [60]

Two specific service scenarios were created: a pick-up service scenario (Scenario 1) named Deliboy and a public shuttle scenario (Scenario 2) named Doby. In both scenarios, the vehicles’ behavior is to approach, stop, talk, and wait. However, each scenario is different in its environment, context, and narrative dialogue of the AV depending on the characteristics of the service. A pick-up service is a commercial service for individuals that focuses on delivering goods efficiently. In the case of Scenario 1, an AV that provides pick-up service, Deliboy, freely travels on a relatively narrow route to deliver goods. Thus, we set the environment and context as follows: the AV stops at a pickup station on a lawn along a sidewalk while a pedestrian waits for their friends near the station. In addition, assuming that the pedestrian is unclear of the tasks the AV is performing, a brief speech of AV was added to inform its situation and ask the situation of the pedestrian. As the AV approaches the pedestrian, it informs what it is doing with a brief greeting (Table 1). On the other hand, unlike Scenario 1, a shuttle service is a public service aimed at groups of people that pursues stability because people are on board. In the case of Scenario 2, it is assumed that a public transportation service, Dovy, encounters a pedestrian who is waiting for another bus. This means that, unlike Scenario 1, the AV and pedestrian are sharing a common denominator. In this context, the pedestrian is at the bus stop at the intersection waiting for a bus. Dovy stops by a nearby Dovy station. Dovy then informs the pedestrian that is a driverless public shuttle and tries to ask more specific questions than Deliboy (Table 1).

In the experiment, there were two non-verbal social cues as manipulated variables: eye movement and conversational distance. The control group was divided into yes (1) and no (0), near (1) and far (0), with the variables varying in each scenario. Additional details on the manipulation of non-verbal social cues are provided in Table 2 (4th and 5th column). All the stimuli were created for the VR environment to simulate the reality of the scenarios [42,61]. These stimuli contents can be fonund in Supplementary Materials.

The AV for Scenario 1 (Deliboy) was designed referring to the design of ‘Delivery Droid’ by Christina Boras [62]. To evoke the image of a pick-up service, a red color and props of a cap and bow tie were applied. The design of AV for Scenario 2 (Dovy) was inspired by ‘Groov’, an AV for public transport by Giulio Urisari [63]. The eyes of the AV were placed on the side, for pedestrians are mostly exposed to the side of such a vehicle. In both cases, the logo was attached to the body as a stimulus to give an appropriate brand experience.

#### 3.1.2. Manipulation Check

In this study, a preliminary investigation of operation inspection was conducted to confirm whether the stimuli were properly designed. Seven students, office workers, and freelancers participated in the preliminary survey. They were shown eight videos developed as experimental stimuli to the survey subjects and were asked to evaluate the stimuli on three criteria: degree of (1) distinguishability among the videos, (2) mediocrity of the voice of AV, (3) association of a particular brand. As a result of the manipulation check, all members distinguished the stimuli according to the conversational distance and the movement of the eyes. Most of the participants reported that the voices of AV were ordinary. The majority of the participants also made remarks that the vehicles were not associated with specific brands. Therefore, the results of the manipulation check, as stated above, indicated a proper design of stimuli. 

### 3.2. Experiment

#### 3.2.1. Participants

Participants of the experiment were all voluntarily recruited from 6 December to 29 December 2021, through online community service for college students in South Korea. A total of 45 people were recruited, and all participated in the experiments and interviews until the end without giving up. As an empirical study, we collected multiple response data from everyone. We used a 2 × 2 within-subjects test in each scenario and got 45 × 2 × 2 = 180 experimental runs. Such a number exceeds the minimum number of runs of 44 to reach the power of 0.8 to conduct repeated measures ANOVA according to the result of power analysis by MorePower 6.0 [64,65]. This data point was also appropriate to qualify the sample size to implement partial least squares structural equation modeling (PLS-SEM) with our model [66,67].

When it comes to the homogeneity of participants’ characteristics, we use the ethnographic approach constructed by Hoff and Bashir [68]. This approach figures out the concept of trust that precedes the interaction process as a structure in which three dimensions are accumulated: dispositional; situational; initial learned. In order to control the trust in situational and initial learned aspects, all participants were allowed to participate in the experiment in the same setting, and information on underlying technology and service background was sufficiently explained in advance. Pertaining to dispositional trust, we identified the sub-elements for this concept (i.e., culture, age, gender, and personality traits like openness) as important characteristics of the study participants, and accordingly, the baseline table is presented as shown in Table 3. Participants were limited to people aged 18–34 considering their inclination to be more positive towards automated vehicles [69]. The participants consisted of 16 men and 29 women. All of them were citizens of South Korea and undergraduate or graduate students. This means that they have the same cultural background.

#### 3.2.2. Process

The experiment was carried out in three stages. Prior to the main experiment, participants were instructed on the overview of the experiment and interview through the consent form. Participants were also informed of the recording of their remarks during interviews, the specific use of the records as well as the removal of them after the use. 

**Figure 2:**
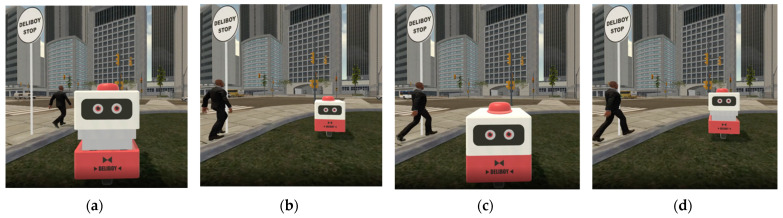
Design of videos within Scenario 1 (**a**) 1A: eye movement yes (1) and conversational distance near (1); (**b**) 1B: eye movement no (0) and conversational distance far (0); (**c**) 1C: eye movement no (0) and conversational distance near (1); (**d**) eye movement yes (1) and conversational distance near (1).

**Figure 3:**
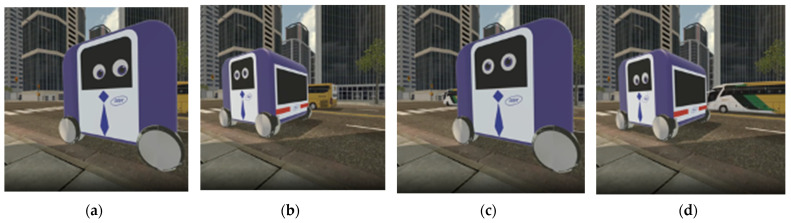
Design of videos within Scenario 2 (**a**) 2A: eye movement yes (1) and conversational distance near (1); (**b**) 2B: eye movement no (0) and conversational distance far (0); (**c**) 2C: eye movement no (0) and conversational distance near (1); (**d**) 2D: eye movement yes (1) and conversational distance near (1).

Prior to the main experiment, participants were instructed that they can freely wander around, take a closer look at or even reach out to the AVs. This was to let the participants immerse themselves in the stimuli. By doing so we intended to capture the response closest to the moment of interaction. In the main experiment, participants wore a VR headset and were presented with eight videos, specifically four for each scenario (Figure 2 and Figure 3). Participants were given a minute to rest in between the watching to offset the unintended effect of the video previously watched. Both the order of scenarios g and the videos within each scenario were randomly assigned. Participants filled out a questionnaire after each video. 

### 3.3. Measurement

A total of 19 items were developed utilizing the validated measures from the prior studies [24,29,70] to measure intimacy, trust, and brand attitude. All items were used after modifying the terms according to the purpose and context of this study. Six researchers participated in the process of selecting items, translating them into Korean as well as co-reviewing them. Specific items for the measurement of each construct are provided in Table 4.

#### 3.3.1. Intimacy

For the measurement items of intimacy, Lee and Choi referred to the study of Berscheid et al., and used six questions after modifying them in accordance with the context of interaction with the conversational agent [29,33]. In this study, the term was changed according to the context of automatic vehicles. All questions were measured using a 7-point Likert scale (1: not at all, 7: very much so).

#### 3.3.2. Trust

Scale items developed by Madsen and Gregor measuring ‘Perceived Reliability’ and ‘Perceived Technical Competition’ for the system were referred to [71], and 6 items were used for trust measures after filtering out similar questions. All questions were measured using a 7-point Likert scale (1: Not at all, 7: Very much so).

#### 3.3.3. Brand Attitude

Brand attitude was measured using items developed by Hwang and Hyun [70]. A semantic differential scale was used (Unfavorable/Favorable, Negative/Positive, Dislike/Like, Bad/Good, Unpleasant/Pleasant, Unsatisfactory/Satisfactory) and all items were measured on a 7-point scale.

#### 3.3.4. Validity and Reliability

Validity and reliability of the items were tested on the full sample as well as within-subject factor (i.e., eye movement and conversational distance) conditions, respectively, using SmartPLS 3.0. Measurements for intimacy, trust, and brand attitude were extracted down to three, three, and five items, respectively, eliminating items with factor loading below 0.70 in any conditions. The AVE (Average Variance Extracted) value of all the remained items was over 0.50, indicating the qualification of convergent validity [67]. Cronbach’s alpha and composite reliability for all remaining items were above 0.70, showing that internal consistency reliability is met. Finally, discriminant validity for the remained questionnaires was tested using the HTMT (Heterotrait-Monotrait Ratio of Correlations) value. HTMT values for every questionnaire did not exceed 0.90 indicating a satisfactory result [72]. Every value for Stone–Geisser’s Q^2^ [73,74] within each scenario case exceeded 0 indicating the predictive power of the model for the latent variables [75]. All the values discussed above were provided in Table A1, Table A2, Table A3, Table A4, Table A5, Table A6, Table A7, Table A8 and Table A9 in Appendix A.

### 3.4. Data Analysis

#### 3.4.1. Quantitative Analyses

Quantitative data analyses were conducted in two steps. First, a repeated measure ANOVA (Analysis of Variance) was conducted through SPSS 26 to examine the effect of non-verbal social cues (i.e., eye movement, conversation distance) on the perception of intimacy, trust, and brand attitude. During the first stage, 3-way repeated measure ANOVA was conducted in advance to test the difference in the perception of intimacy, trust and brand attitude according to the scenario (pick-up service vs. public shuttle). Second, an analysis of the structural equation modeling (SEM) via SmartPLS 3.0 was implemented to explore how the relationships between intimacy, trust, and brand attitude vary depending on the social cues. SmartPLS is a widely used software for validating the structural model with partial least squares path modeling (PLS-SEM). PLS-SEM is a comparatively flexible method in that the approach is less affected by sample sizes, distribution of data, and complexity of the model [47].

#### 3.4.2. Qualitative Analyses

Qualitative analyses were performed to multidimensionally understand the main phenomena experienced by the participants. The analyses were conducted with the transcripts of interviews. We repeated the process of elaborating and categorizing conceptual codes extracted through the continuous comparative analysis to gain insight into the experience of the participants based on ‘the constant composite method’ [53].

The analyses process was implemented in three stages. In the first stage, the authors of this study repeatedly read raw data together and performed open coding in which units of contents to be meaningful were extracted, conceptualized, categorized, and labeled [76]. In this stage, concepts (i.e., assignment of codes to meaningful conditions, phenomena, events, etc.) and categories (i.e., groupings of concepts in homogeneity) are formed [77]. After the first stage, axis coding was performed: the categories were arranged and re-categorized according to a specific axis and frame [78]. This work makes it possible to effectively understand the relations among the categories [20]. In this study, categories were classified into causal conditions, phenomenon, contextual conditions, intervention conditions, action/interaction strategies, and results proposed by Corbin and Strauss [76]. Finally, a visual model was developed based on the analyses of the phenomena using paradigm analysis.

This study utilized several strategies to meet the validity and reliability of the qualitative analyses. First, four coders participated in the analyses, and the conversational validity proposed by Mills was satisfied through the repetitive process until full agreement was reached [71]. Next, peer examination proposed by Merriam was used [79]. Peer examination is a method to request a review of research data and analysis results from fellow researchers with qualitative research experience. In this study, a review was requested by a researcher who was not a direct participant in this study and held a master’s degree with experiences of a qualitative study. In addition, conceptualization work closely related to the results of quantitative analyses was performed, and the comprehensive interpretation and discussion were described after the analyses. This means that the result validity suggested by Mills is satisfied by delivering answers to the research questions and achieving the research aim [71].

## 4. Results

### 4.1. Results of Quantitative Analyses 

#### 4.1.1. Effect of Non-Verbal Social Cues on Intimacy, Trust, and Brand Attitude

**Figure 4:**
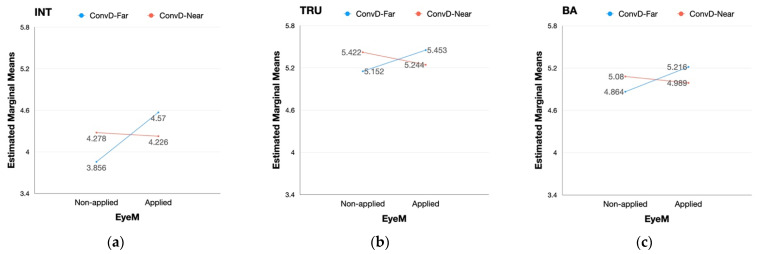
Full sample: interaction effect between eye movement (EyeM) and conversational distance (ConvD); (**a**) interaction effect on intimacy; (**b**) interaction effect on trust; (**c**) interaction effect on brand attitude.

Prior to conducting repeated measures of ANOVA, normality was tested for each sub-group of the two scenarios. Tests of sphericity were omitted since the level of repeated measures variables (i.e., scenario type, eye movement, conversational distance) was less than three [80]. Every sample of the sub-groups showed normality in distribution as the value of skewness ranged between −2 and +2 while the value of kurtosis ranged between −7 and +7 [55,81]. Repeated measures of 3-way ANOVA were implemented to test the difference in the effect of scenario type, eye movement, and conversational distance on the perception of intimacy, trust, and brand attitude. A summary of the results is reported in Table A10 of Appendix A. Both the type of scenario and application of eye movement had a significant influence on the perception of intimacy while the rest of the variables were not affected by the factors. However, conversational distance did not have any significant effect on intimacy, trust, and brand attitude. Interaction effects between the factors were also tested. Among the four types of interactions, only the interaction between eye movement and conversational distance was found to have a significant effect on intimacy, trust, and brand attitude. Detailed appearances of the interaction effect on each dependent variable are provided in Figure 4.

To take a closer look at the effect of two factors of non-verbal social cues within each scenario, repeated measure ANOVA was also conducted for each scenario type. The main effect of two factors of social cues (i.e., eye movement, conversational distance) on the perception of intimacy, trust, and brand attitude was found to be partially valid in the Scenario 2 group. The application of eye movement was discovered to have a valid effect on the perception of intimacy in the scenario of a public shuttle. 

**Figure 5:**
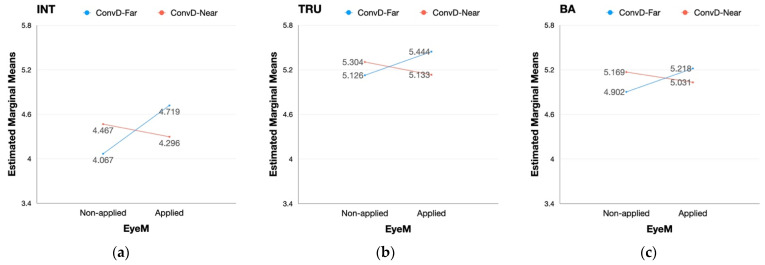
Pick-up service scenario: interaction effect between eye movement (EyeM) and conversational distance (ConvD); (**a**) interaction effect on intimacy; (**b**) interaction effect on trust; (**c**) interaction effect on brand attitude.

Unlike the partial main effect, the interaction between eye movement and conversational distance had a significant effect on every dependent variable (i.e., intimacy, trust, brand attitude) in both scenarios as provided in Table A11 and Table A12 of Appendix A. The interaction between eye movement and conversational distance had a similar effect on every dependent variable in the case of pick-up service (Figure 5). With the eye movement static, the degrees of each variable were reversed with eye movement applied while the degrees of each variable were lower when the distance was set far than when the distance was close. Specifically, when the eye movement was applied, the degrees of each variable decreased when the conversational distance was set close while those of each variable increased when the conversational distance was far between the pedestrian and the vehicle (Figure 5). 

**Figure 6:**
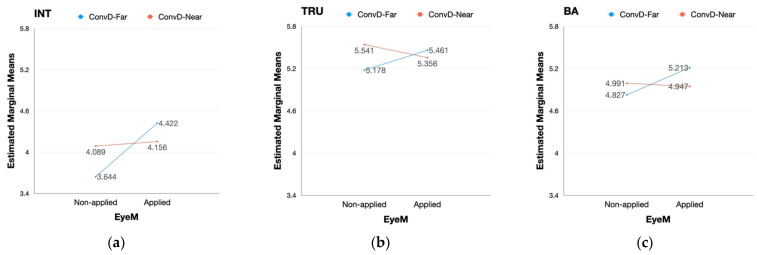
Public shuttle scenario: interaction effect between eye movement (EyeM) and conversational distance (ConvD); (**a**) interaction effect on intimacy; (**b**) interaction effect on trust; (**c**) interaction effect on brand attitude.

Contrary to the case of a pick-up service, the interaction between the two factors of social cues showed different aspects in the effect on intimacy, trust, and brand attitude in the case of the public shuttle. The perception of intimacy was lower when the conversational distance was far than when the distance was close without eye movement. However, the value was reversed when eye movement was applied: intimacy was higher when conversational distance is far than when the distance is close (Figure 6a). Unlike intimacy, the interaction effect on trust and brand attitude appeared in different forms. The degrees of both dependent variables decreased when the eye movement was applied with the conversational distance set close between the pedestrian and the autonomous vehicle. However, the degrees of trust and brand attitude increased when the eye movement was applied with a conversational distance set far between the two entities (Figure 6b,c). The values of trust and brand attitude with conversational distance set far excelled those of the two dependent variables with conversational distance set close when eye movement was applied.

#### 4.1.2. The Relationships among Intimacy, Trust, and Brand Attitude

**Figure 7:**
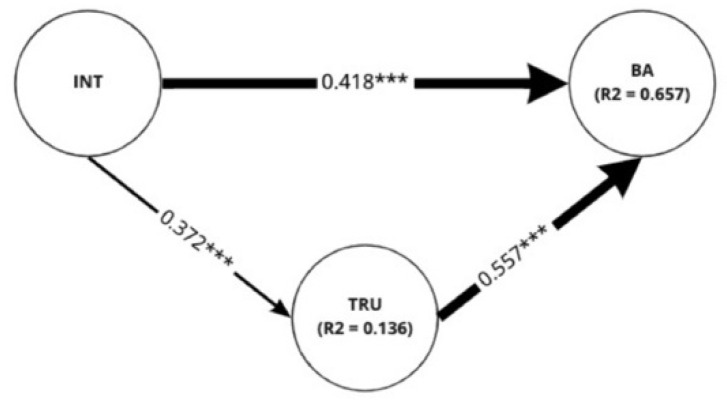
Full sample: Results of analysis on the structural model for the full sample (N = 45). R^2^ values indicated in the figures are adjusted values of R^2^. *** *p* < 0.001.

Structural equation modeling was utilized to examine the relationships between intimacy, trust, and brand attitude. To elucidate the varying relationships among the latent variables (i.e., intimacy, trust, brand attitude) regarding the social cues, structural models of every sub-group within each scenario type were also analyzed. We conducted bootstrapping with 5000 resamples for the *t*-test [81]. Figure 7 demonstrates the results of the analysis of the full sample. It was revealed that intimacy both directly (β = 0.418, *p* < 0.001) and indirectly (β = 0.207, *p* < 0.001) affected brand attitude in a positive manner. For the indirect effect, intimacy influenced brand attitude through the mediation of trust. Intimacy directly affected trust with a path coefficient of 0.372 (*p* < 0.001) while trust directly influenced brand attitude with a path coefficient of 0.557 (*p* < 0.001). 

**Figure 8:**
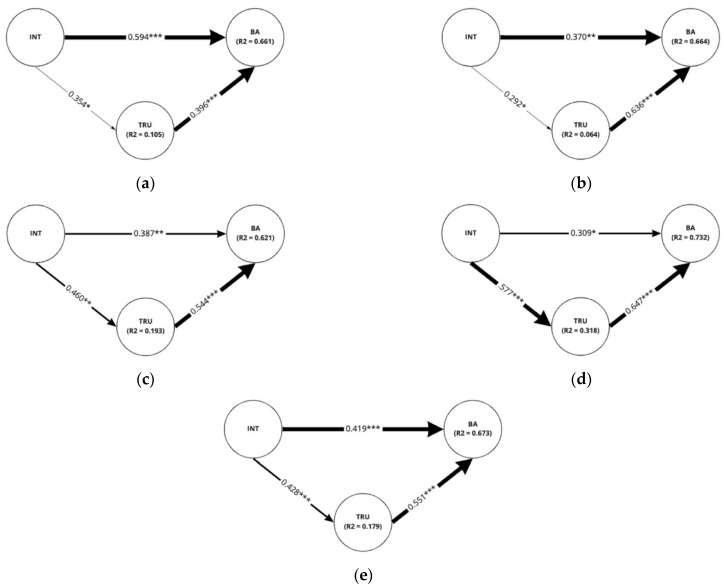
Pick-up service scenario: Results of analysis on each structural model for the application of eye movement and conversational distance (**a**) eye movement applied and conversational distance close; (**b**) eye movement unapplied and conversational distance far; (**c**) eye movement unapplied and conversational distance close; (**d**) eye movement applied and conversational distance far; (**e**) full sample for the scenario. INT: Intimacy, TRU: Trust, BA: brand attitude. R^2^ values indicated in the figures are adjusted values of R^2^. * *p* < 0.05, ** *p* < 0.01, *** *p* < 0.001.

In the scenario of a pick-up service, the structural model showed the same relationships among intimacy, trust, and brand attitude regardless of the different application of social cues (Figure 8). However, the strength of the direct effect of trust on brand attitude and the indirect effect of intimacy on brand attitude differed among groups partially as provided in Table 5. Trust was found to have a stronger influence on brand attitude when ‘eye movement was not applied, and the conversational distance was far between the two entities (β = 0.636, *p* < 0.001)’ or when ‘eye movement was applied but the conversational distance was far (β = 0.647, *p* < 0.001)’ than when ‘eye movement was applied, and the distance was set close (β = 0.396, *p* < 0.001)’. Furthermore, the indirect effect of intimacy on brand attitude mediated by trust was stronger when ‘eye movement was applied but the conversational distance was far (β = 0.373, *p* < 0.001)’ than when ‘eye movement was applied, and the distance was close between pedestrian and the autonomous vehicle (β = 0.140, *p* < 0.05)’. 

**Figure 9:**
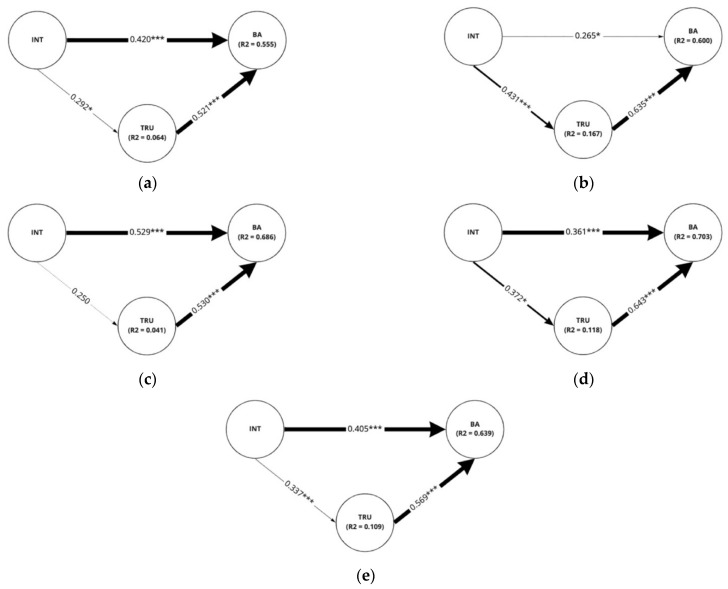
Public shuttle scenario: results of analysis on each structural model for the application of eye movement and conversational distance (**a**) eye movement applied and conversational distance close; (**b**) eye movement unapplied and conversational distance far; (**c**) eye movement unapplied and conversational distance close; (**d**) eye movement applied and conversational distance far; (**e**) full sample for the scenario. INT: Intimacy, TRU: Trust, BA: Brand Attitude INT: Intimacy, TRU: Trust, BA: Brand Attitude. R^2^ values indicated in the figures are adjusted values of R^2^. * *p* < 0.05, *** *p* < 0.001.

When conducting analyses on the sub-groups within Scenario 2, both intimacy and trust had a significant positive effect on brand attitude as shown in Figure 9. Intimacy had a positive influence on trust in all cases for the application of social cues except for the case when ‘eye movement not applied while the conversational distance set to close’ (Figure 9c). In such a case, only the direct positive effect of intimacy (β = 0.529, *p* < 0.001) and trust on brand attitude (β = 0.530, *p* < 0.001) was found valid while the indirect effect of intimacy on brand attitude and the direct effect of intimacy on trust was found insignificant. Moreover, unlike Scenario 1, the difference between the degree of significant effect in each case was revealed to be insignificant indicating that each corresponding effect is similar in strength. 

### 4.2. Results of Qualitative Analyses

The main purposes of the analyses were to discover conceptual categories affecting the emotional perception of pedestrians in each scenario and to understand the complex relations among the concepts. Thus, we explored the participants’ experiences before and after the interaction and their ordinary thoughts on self-driving cars and related services. A total of 42 meaningful concepts were discovered, and 16 conceptual categories were developed after similar concepts were grouped and reconstructed. Table 6 shows the attributes and dimensions of each concept.

‘Pursuing Context Combination’ and ‘Maintaining Context Separation’ are causal conditions that lead to the key phenomena. Several participants emphasized that they were just passing by or unrelated people while watching the scenario video. However, when an AV approached and tried to get into conversation with pedestrians, they were perceived as actors breaking the conceptual boundaries drawn between separate contexts of AV and pedestrians. 

The tug-of-war between AV ‘seeking contextual combination’ and pedestrians ‘seeking to adhere to the state of separation’ took place under the contextual conditions of ‘Physical Characteristics’ and ‘Environmental Characteristics’. These contextual conditions had close connections to the conversational distance and were perceived differently by scenarios. Despite the numerically controlled distance between pedestrians and vehicles, several participants said that the AV in Scenario 1 approached in a much more burdensome way than the AV in Scenario 2. Participants remarked that Dovy was a more stable being because it is larger in shape and gives less of a sense of speed compared to Deliboy. This indicates that participants were influenced by the ‘Physical Characteristics’ such as the size and speed of AV. In addition, the operating environment of AV in the scenario was also a significant factor in terms of perception of AV. Dovy was considered a monorail moving on a set road, therefore was considered a predictable and stable object. On the contrary, Deliboy was perceived as an unstable being wandering around the area as it crossed the boundaries between the sidewalk and the lawn.

The central phenomenon created by the combination of causal and contextual conditions was ‘Conceptual Definition of the Space’. This conceptual category refers to a co-existing space of the AV and pedestrians that is contextually pictured and defined in the pedestrian’s mind according to the observed physical and environmental properties of the vehicles. The noticeable part was that participants understood variables such as eye movement or conversational distance within a continuous frame rather than recognizing them in fixed or segmented scenes.

The conditions mediating the behavior and interaction strategy taken by pedestrians were found in the central phenomenon. Such conditions were classified into four categories. Diverse categories varying with the participants were developed according to the sub-concepts in each category. (1) The ‘Pre-expected’ category includes attributes such as the degree of expectation (high–low) for the cognitive/judgmental competence of the self-driving cars, the expected interactional attributes (human–mechanical), and the degree of trust required for the services (high–low). (2) The degree of familiarity (high–low) with the service is presented as an attribute of the category ‘Pre-familiarity’. (3) The ‘Spare Time’ category includes the concept that pedestrians’ attitudes toward the formation of relationships with autonomous vehicles vary depending on the urgency of the encountered moment with the vehicles. (4) ‘Empirical Metaphor’ is a factor that explains a phenomenon affecting behavior or interaction strategies when pedestrians come up with a metaphor from a specific experience during the interaction. 

Participants in the study also showed various action/interaction strategies. First, ‘Task-oriented Evaluation’ was the main strategy. It refers to the evaluation of vehicles according to the necessary tasks assigned to them. Some participants perceived humanness from the social behavior of AV, especially from the blinking. Some even felt the voices to be more familiar than the vehicles blinking and trying to narrow the conversational distance. Since the voices of the vehicles were controlled, such experiences allude that the visual experiences were transferred to auditory experiences. Furthermore, some people recognized the sociality of self-driving cars as a function itself. Distinguishing cognitive experiences observed from this group are as follows: (1) Regarding the vehicles’ attempts to communicate as signs of the possibilities to control them. (2) Recognizing the gazing as an act of keeping an eye on all sides during the drive. (3) Paying attention to the motor function of Deliboy freely crossing the border to approach. (4) Noticing the vehicles’ abilities to accurately locate the pedestrian. (5) Judging that the vehicles are delicately designed, for they are equipped with sociality. 

**Figure 10:**
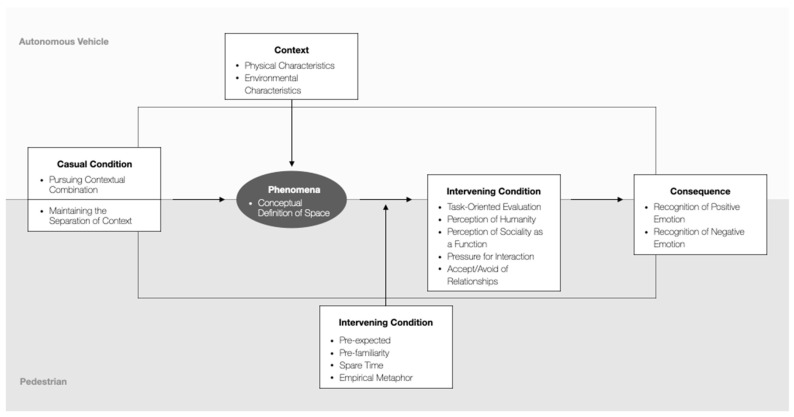
Conceptual model of paradigm analyses.

Finally, most of the participants tended to avoid the relationship with the vehicles. Some participants reported that they felt pressure to interact while some said they perceived relations with AV or felt that AV were equal beings. It should be noted that the participants felt negative emotions such as instability, pressure, distance, and threat rather than solely experiencing positive emotions like intimacy and trust from the friendly approach of self-driving cars. Figure 10 is presented as a conceptual model summarizing the paradigm analyses illustrated above.

## 5. Discussion

### 5.1. Consideration of Service Context

In this study, differences in the degree of intimacy were found for each scenario indicating that the characteristics of the service can affect the formation of an intimate relationship. When repeated measures of 3-way ANOVA were implemented to test the difference in the effect of scenario type, eye movement, and conversational distance, the type of scenario had a significant influence on the perception of intimacy while the others were not affected by the factors. In the case of a pick-up service, intimacy measured higher than in the case of the public shuttle. Such difference was due to participants’ tendency to consider Deliboy small, cute, and friendly. It can be concluded that the appropriate size of the body of AV to deliver food quickly induces intimacy towards the vehicle. Furthermore, participants had the pre-expectation that the public shuttle had to provide formal service rather than forming an intimate relationship. This alludes that such a pre-expectation inhibited pedestrians to feel close to Dovy.

It was confirmed that the same social cues and their interactions can generate different effects depending on the type of service. The perception of intimacy did not decrease but increased, even with the AV set closer to the pedestrian with the application of eye movement, while the degrees of other variables (i.e., trust, brand attitude) were found to decrease due to the interaction effect of social cues. The strong effect of eye movement on intimacy in Scenario 2 gives a hint. When repeated measures of ANOVA were conducted for each scenario type, eye movement had a valid effect on the perception of intimacy only in the case of the public shuttle. In the interview, for the participants considering physical characteristics as a crucial condition, the movements of eyes were found to be more dramatic in the Dovy case due to its large body and eye. 

In the perception of trust, the tendency of the interaction effect was similar between the two scenarios, but the highest case was different. In the case of pick-up service, the highest was when the distance was set far but eye movement was applied. It was, however, found that the highest perception of trust was when the conversational distance was set close and eye movement was not applied in the scenario of the public shuttle. Furthermore, such a case was the only one where intimacy had an insignificant effect on trust. That is, trust in this case was not formed by intimacy. This result can be interpreted as originating from ‘Trust Level Required by Service’ and ‘Pre-familiarity’. In the interview, the participants made remarks that the level of trust should be high for buses that they had to board themselves. A bus coming to stop right in front of pedestrians was a typical ‘Empirical metaphor’ of the public transportation service participants had experienced for a long time. Therefore, the credibility of the typicality would have led them to comparatively underestimate the influence of intimacy on trust, especially in the case of the public shuttle. In the case of the other three design types in Scenario 2, however, the development of intimacy was revealed significant to form trust. When the conversational distance was far, the application of eye movement was found crucial in recognizing the AV’s attempt to ‘Pursue Contextual Combination’ and form relationships with pedestrians.

### 5.2. Sociality vs. Task

According to the results of the structural equation modeling for each scenario, building intimacy in both scenarios was confirmed as a valid strategy for forming trust and brand attitude. As a result of the analysis of the full sample, it was revealed that intimacy affected brand attitude in a positive manner directly as well as through the mediation of trust. Considering that the effect of intimacy on trust was not very strong, however, a separate strategy to form trust is required to trigger a positive brand attitude.

In a qualitative study, several participants emphasized that intimacy and trust were both important in determining attitudes towards service providers and brands. However, they also stressed that it was a crucial point whether the AV was performing its task steady in assessing its trustworthiness. The concept that explains this is ‘Task-oriented Evaluation’, which was one of the action/interaction strategies pedestrians took. Participants tried to identify the motion of looking for customers who ordered pizza and considered the social behavior of making eye contact and trying to have small talk unnecessary. In some cases, however, receptive behavior was seen when the social behavior of the vehicles did not deviate significantly from the service context. For instance, in Scenario 2, Dovy asking “which bus are you waiting for?” was in line with the expected task of the AV.

The quantitative and qualitative results of this study indicate that sociality and task are not in opposition, but that it is a matter of balance in which sociality should be delivered according to the task expected from the service. Most of the participants said that excessive sociality reduces intimacy and trust. Deliboy jumping over obstacles to get closer caused participants to worry whether the food inside would turn into a mess. However, an adaptive strategy could be taken, such as narrowing the distance only in a clearly zoned space (e.g., sidewalk). It would be another choice to devise a variety of design methods, such as making a transparent body, to reassure pedestrians that food is being safely delivered.

### 5.3. Psychological Zoning

As stated by the results of repeated measures of ANOVA for each scenario type, the AV’s social behavior did not have a significant main effect on trust and brand attitude in all scenarios. However, the social cues had significant interaction effects on all dependent variables. In the pick-up service scenario, the degree of each variable decreased when the eye movement was applied with the distance set to close. Conversely, the variables were measured higher when eye movement was applied in a state where the distance was set farther. Furthermore, the perception of intimacy, trust, and brand attitude were all measured the highest in the case of AV with eye movement applied to maintain a certain distance from pedestrians. As seen from the above, a slightly different result appeared in the public shuttle scenario while the significance of the interaction effect was the same.

As a result of comparing the detailed paths of all groups in the structural equation modeling, significant differences in the influences of trust on brand attitude were observed in only two cases: between group A (eye movement applied and conversational distance close) and group B (eye movement unapplied and conversational distance far), and between group A and group D (eye movement applied and conversational distance far) in Scenario 1. The differences between A and D suggest that the conversational distance within the context of a delivery service should be deliberately controlled considering its impact when the eye movement is applied. In the same scenario, the invalid difference between group A and group C indicates that once AV is located at a certain distance, pedestrians maintain an equivalent level of trust regardless of the application of eye movement.

From the interaction effect between eye movement and conversational distance, the effect of conversational distance was perceived as stronger in Scenario 1. This was found to be closely related to the ‘Conceptual Definition of Space’ phenomenon found in qualitative analyses. Participants imaginatively drew the path of the AV and predicted the next operation or behavior and perceived the same distance differently. For instance, Deliboy in Scenario 1 came over the border to the lawn where pedestrians stood, and the participants regarded this as an act of actively breaking the boundaries. Some participants felt an immediate threat from such behavior while others considered the same behavior as a clever behavior accurately identifying the locations of pedestrians. Participants evaluated the stability of the vehicles and formed their initial trust in AV based on their imaginations about the given space. Regarding this psychological description, designers can give a sense of stability to the pedestrian through visible indication. We suggest psychological zoning as a specific method of visual communication: clearly indicating the potential area for the AV to stop or the path of the AV on the floor using a brand color. 

### 5.4. Comprehensive Discussion for Limitation and Future Work

Despite the results and significance of this study, it has methodological and scope-related limitations.

First, considering methodology, there is a limitation of fully controlling the influence of accumulated familiarity as the experiment participants repeatedly evaluate their experiences with driverless services. In this study, to overcome this limitation, the order of interaction with the videos was randomized for each study participant, and the statistical validity was met. The emotional response, however, that could emerge from the repetition was not sufficiently explored.

Another issue regarding the methodology is the sample size in terms of statistical validation and generalization of our study findings. Previous studies in the human–AV interaction field have been conducted within the context of social and public acceptance considering trust or other perception variables (i.e., perceived risk and safety) and the number of participants varies depending on the purpose and method of the study. Their study category can be divided into three areas: (1) opinion framework (2) perception modeling (3) design proposal.

Opinion framework studies investigate general knowledge, affection, and opinion about autonomous vehicles and develop conceptual factors or constructs. We sub-categorized them into three types according to the stimulation method (i.e., none of stimuli, text description, field simulation). Some studies have their participants freely introduce their usual thoughts. Schneble and Shaw explored how the public defines AVs in terms of advantages, disadvantages, and reliability through semi-structured interviews (16 people) [82]. Lokshina’s research team implemented an online survey for 208 people to illuminate the factors that influence AV users’ initial trust [83]. Others included text descriptions about AVs as a stimulus. A structured in-depth interview case for 25 people was conducted to study the motivational structure of engaging in autonomous driving [84]. Most large-scale online survey studies use text descriptions to focus on their targeting autonomous services. The studies that we analyzed for this category have study participants which span from 192 to 1582 people [85,86,87,88]. Recently, to induce participants more absorbed in the scenario, the Field Operational Test (FOT) was introduced [89,90]. In the two FOT studies, study participants are observers within a scenario which is as if it were happening in the real world without any intervention by a researcher. After that, some observers are selected randomly as interviewees (11 and 19 people, respectively) to share their opinion as to the experience. In this setting, researchers explicit the latent concepts in the form of theoretical discussions.

In our in-depth discussion of the previous literature, perception modeling studies use manipulated and controlled text descriptions. For example, Waung et al. examine the effect of manipulation of information type involved in the descriptions on perceived AV performance risk, trust in AV performance, and intention to use AVs through an online survey of 337 people [91]. Hegner’s team carried out a survey investigation of 369 people manipulating a text-based scenario for structural analysis considering factors that influence prior intention to adopt [86].

Last, the category of design proposal studies assigns a task to their experiment participants and analyses one’s behaviors to test their design prototype [61,92]. In such experimental settings, physical interface (i.e., handle and cycle) and video stimuli viewed through tablet PCs or VR headsets were adopted. In the case of tablet PCs, 98 people were studied while 18 people with VR headsets repeatedly evaluate multiple designs.

Thus, in our hybrid study combining survey and interview, 45 people as in-depth interviewees are enough to examine the experience and opinion and to construct a conceptual framework like Figure 9. Compared with the FOT methodology, we provided our participants with an absorbing environment that was more immersive than tablet PCs, but less than a real-world production. However, it should be considered that we need to enlarge the sample size and assign a task to observe participants’ behavior to generalize the perception model analysed in our statistical studies. Although we provided the opportunities of freely wandering around, taking a closer look at, or even reaching out to the AVs to observe participants’ behaviors and to ask questions in the interview section, we have no concrete task or analysis process or protocol. So, in our future work, behavior analyses in quantitative and qualitative manners should be conducted in an interactive task setting with a larger size of sample about 100 people when referring to the study of Lee and Lee [92].

With enlarging the sample size, we expect a more sophisticated analysis to be possible. In this study, discussions were conducted based on interaction effects between manipulated variables, but a study with a larger sample will further explore other factors associated with the effects of design factors and attitudes toward driverless services. Specifically, the democratic characteristics and personality traits of participants can be illuminated as independent variables. In the current study, the gender of participants brings to the surface the homogeneity of the baseline population. Gender had statistical significance to intimacy with eye movement and far-distance (t = −2.229, * *p* < 0.05). This calls attention to the issue of the possible influence of gender on attitudes toward AV [69].

Despite the limitations in variable control, this study was aware of the context factors in qualitative analyses. Future studies of quantitative analysis should be implemented to concretely explicate the influence of such variables on the attitude toward AV. Furthermore, subsequent studies with the context of pedestrians in the crowd need to be addressed. The psychology of pedestrians in the crowd can present other research questions with the presence of others as an environmental factor. Further insights into the individual attitude could be obtained with a study on pedestrians’ responses in a real context.

## 6. Conclusions

This study explored the social interaction and relationship between AVs and pedestrians. Specifically, we studied communication design methods for the formation of pedestrians’ trust and attitudes toward autonomous vehicles using non-verbal social cues.

First, we examined the manipulation effect of non-verbal social cues statistically. As a result of the ANOVA analyses, the interaction effect created by non-verbal social cues (i.e., eye movement, conversational distance) had a significant effect on every dependent variable (i.e., intimacy, trust, brand attitude) and the pattern was different depending on the scenario. According to the structural equation modeling, intimacy both directly and indirectly has significant effects on brand attitude in a positive manner. For the indirect effect, intimacy influenced brand attitude through the mediation of trust. Differences between groups in subjective perception and response were identified. Trust has a stronger influence on brand attitude when ‘eye movement was not applied, and the conversational distance was far between the two entities’ or when ‘eye movement was applied but the conversational distance was far’ than when ‘eye movement was applied, and the distance was set close’. Furthermore, the indirect effect of intimacy on brand attitude mediated by the trust was stronger when ‘eye movement was applied but the conversational distance was far’ than when ‘eye movement was applied, and the distance was close between pedestrian and the autonomous vehicle’. 

Further, quantitative and qualitative results were synthesized to discuss considerations according to the service context, and task-related methods and psychological zoning to effectively utilize sociality were presented with specific design examples. These findings are differentiated in that they are the results of investigating instantaneous and subjective impressions in interactions between AVs and pedestrians, which have not been sufficiently discussed before.

This study provides academic, practical, and social contributions. We proposed models and factors that could be further developed by pedestrian–AV studies. Furthermore, Findings of the current research provide communication designers with insightful strategies. Finally, we could make an impact on the social acceptance of AV-based services.

## Figures and Tables

**Table 1:** Overview of Scenario 1 and Scenario 2.

	Scenario 1: Pick-Up Service, Deliboy	Scenario 2: Public Shuttle, Dovy
Vehicle’s Behavior	Approach-Stop-Talk-Wait	
Environment	Lawn along sidewalk	A bus stop at the intersection
Context	You are waiting for your friend near the pickup station where Deliboy stops. You are not the orderer of the food.	You are at the bus stop to catch a bus. Before your bus arrives, Dovy stops by a nearby Dovy Station.
Speech	“Hi! I am Deliboy. I am supposed to meet with the person who ordered pizza here, but they are not here yet. What were you up to?”	“Hello! This is Dovy, a self-driving shuttle bus service. Which bus are you waiting for?”

**Table 2:** Overview of stimuli.

Visual Social Cue	Control	Social Signals	Scenario 1: Pick-Up Service, Deliboy	Scenario 2: Public Shuttle Bus, Dovy
Eye Movement	Yes (1)	Vehicle regarded as trying to make eye contact with its eye movement	Eyes blinking and body adjusting to make eye contact with a pedestrian	Eyes blinking and moving to make eye contact with a pedestrian
	No (0)	Vehicle regarded as not sending recognizable social signals without eye movement	Eyes not blinking and body staying still	Eyes not blinking and without any movement
Conversational Distance	Near (1)	Vehicle actively approaching within the ‘personal zone’ of the pedestrian for conversation	Keeping a distance of 1 m	Keeping a distance of 1 m
	Far (0)	Vehicle carefully keeping a distance in the ‘boundary of social zones’ for communication	Keeping a distance of 3.5 m	Keeping a distance of 3.5 m to the left

**Table 3:** Baseline characteristics of participants (N = 45).

Characteristics	Participants (*n* = 45)
Age, median (IQR)	23 (21; 26)
Gender, *n* (%)	
Male	16 (35.5)
Female	29 (64.5)
Country of Residence, *n* (%)	45 (100)
South Korea	

**Table 4:** Constructs and measurement items (X refers to the service name).

Construct		Measurement Items	Sources
Intimacy (INT)		I became familiar with X	[29,33]
		X will affect my choice of the service	
		I feel X is emotionally close to me	
		I feel like X is my close friend	
		I feel familiar with X	
Trust (TRU)	Perceived Reliability	X will always perform tasks consistently	[24]
		I believe that X will work properly	
		X acts trustfully	
	Perceived Technical Competence	X will have sufficient knowledge of what X has to do	
		X will be able to provide quality services as well as people who provide the same service	
		X will use appropriate methods to make judgments	
Brand Attitude (BA)		I am not satisfied with X	[70]
		I think X is unpleasant/I think X is pleasant	
		I think X is bad/I think X is good	
		I do not like X/I like X	
		I am negative/positive about X	
		I am not in favor of X	

**Table 5:** Multi group analysis for pick-up service scenario 1 (N = 45).

Path	Path Coefficient (1A, 1B)	Path Coefficient (1A–1D)
TRU → BA	−0.240 *	−0.251 *
INT → TRU → BA	-	−0.233 *

Only significant values were reported. 1A: Eye movement applied and conversational distance close. 1B: eye movement unapplied and conversational distance far. 1D: eye movement applied and conversational distance far. INT: Intimacy, TRU: Trust, BA: Brand Attitude. * *p* < 0.05.

**Table 6:** Results of Qualitative Analyses.

Concept	Category	Property	Dimension	Aspect	Paradigm
Crossing the Boundary	Pursuing Contextual Combination	Degree of Pursuit	Active–Passive	Autonomous Vehicle	Casual Condition
Starting the Conversation					
Sharing the Situation					
Separation of Context	Maintaining the Separation of Context	Degree of Pursuit	High–Low	Pedestrian	
Bystander					
Size	Physical Characteristics	Size	Big–Small	Autonomous Vehicle	Context
Mobility		Movement Stability	Stable–Unstable		
Environmental Characteristics	Environmental Characteristics	Degree of Restriction of the Driving Environment	High–Low	Autonomous Vehicle	
Conceptual Path	Conceptual Definition of the Space	-	-	Pedestrian-Autonomous Vehicle	Phenomena
Perceived Distance					
Expected Capability	Pre-Expected	Degree of Expectation of its Capability	High–Low	Pedestrian	Intervening Condition
Expected Interaction		Expected Interaction Characteristics	Human-Like–Machine-Like		
Trust Level Required by Service		Degree of Trust Required by the Service	High–Low		
Previous Information	Pre-Familiarity	Familiarity	High–Low	Pedestrian	
Accumulated intimacy					
Repeated Experience					
Spare Time	Spare Time	Spare Time	Relaxed–Urgent	Pedestrian	
Personal Metaphor	Empirical Metaphor	-	-	Pedestrian	
Familiar Metaphor					
Task-Oriented Observation	Task-Oriented Evaluation	Judgment Based on Its Task	Task-Oriented Evaluation–Social Interaction-Oriented Evaluation	Pedestrian	Action-Interaction Strategy
Importance of Service Context					
Recognition of Unnecessary Social Skill					
Feeling like a human	Perception of humanness	Attitude	Positive–Negative	Pedestrian	
Recognition of Sociality as Manipulation Function	Perception of Sociality as a Function	Attitude	Positive–Negative	Pedestrian	
Recognition of Sociality as Motor Function					
Recognition of Sociality as Cognitive Function					
Recognition of Sociality as Judgement Function					
Recognition of Sociality as a Function of Expressing intimacy					
Pressure to Respond	Pressure for Interaction	Perceived Level of Pressure	High–Low	Pedestrian	
Instant Desire for Interaction					
Perceived as Equal	Accept/Avoid of Relationships	Relationship Acceptance Attitude	Preference for relationship acceptance–Avoidance for relationship acceptance	Pedestrian	
Perception of Relationship					
Conscious Response					
Preference in Avoidance					
Stability	Recognition of Positive Emotion	Perceived Positive Emotion	High–Low	Pedestrian	Consequence
Intimacy					
Trust					
Unstableness	Recognition of Negative Emotion	Perceived Negative Emotion	High–Low	Pedestrian	
Pressure					
Distance					
Threat					

## Data Availability

The data presented in this study are available on request from the first author.

## Supplementary Materials

The following supporting information can be downloaded at: drive.google.com/drive/folders/1qRim137ULiTqadHUvdygEUkLsDMJSsMZ?usp=sharing (accessed on 19 January 2022).

## Appendix A

**Table A1:** Construct Validity and Reliability of Full Sample (N = 45).

Construct	Item	Outer Loading	Cronbach’s Alpha	Composite Reliability	AVE
Intimacy	INT01	0.946	0.935	0.959	0.886
	INT02	0.938			
	INT03	0.940			
Trust	TRU01	0.925	0.907	0.942	0.844
	TRU02	0.924			
	TRU03	0.907			
Brand Attitude	BA01	0.928	0.957	0.967	0.853
	BA02	0.937			
	BA03	0.923			
	BA04	0.908			
	BA05	0.946			

**Table A2:** Construct Validity and Reliability of each case in Delivery Service Scenario.

Case	Construct	Item	Outer Loading	Cronbach’s Alpha	Composite Reliability	AVE
1A	Intimacy	INT01	0.943	0.938	0.960	0.889
		INT02	0.941			
		INT03	0.945			
	Trust	TRU01	0.869	0.816	0.891	0.731
		TRU02	0.853			
		TRU03	0.843			
	Brand Attitude	BA01	0.927	0.939	0.954	0.806
		BA02	0.922			
		BA03	0.870			
		BA04	0.853			
		BA05	0.915			
1B	Intimacy	INT01	0.95	0.953	0.970	0.915
		INT02	0.949			
		INT03	0.970			
	Trust	TRU01	0.929	0.895	0.935	0.827
		TRU02	0.909			
		TRU03	0.890			
	Brand Attitude	BA01	0.918	0.948	0.960	0.829
		BA02	0.923			
		BA03	0.930			
		BA04	0.875			
		BA05	0.906			
1C	Intimacy	INT01	0.947	0.911	0.943	0.846
		INT02	0.905			
		INT03	0.906			
	Trust	TRU01	0.902	0.915	0.947	0.855
		TRU02	0.960			
		TRU03	0.912			
	Brand Attitude	BA01	0.937	0.968	0.975	0.886
		BA02	0.953			
		BA03	0.951			
		BA04	0.922			
		BA05	0.944			
1D	Intimacy	INT01	0.960	0.911	0.943	0.846
		INT02	0.955			
		INT03	0.947			
	Trust	TRU01	0.955	0.915	0.947	0.855
		TRU02	0.939			
		TRU03	0.945			
	Brand Attitude	BA01	0.944	0.968	0.975	0.886
		BA02	0.930			
		BA03	0.944			
		BA04	0.934			
		BA05	0.938			

1A: Eye Movement applied and Conversational Distance close. 1B: Eye Movement unapplied and Conversational Distance far. 1C: Eye Movement unapplied and Conversational Distance close. 1D: Eye Movement applied and Conversational Distance far.

**Table A3:** Construct Validity and Reliability of each case in Public Transportation Service Scenario.

Case	Construct	Item	Outer Loading	Cronbach’s Alpha	Composite Reliability	AVE
2A	Intimacy	INT01	0.960	0.914	0.946	0.854
		INT02	0.955			
		INT03	0.947			
	Trust	TRU01	0.955	0.865	0.917	0.786
		TRU02	0.939			
		TRU03	0.945			
	Brand Attitude	BA01	0.944	0.938	0.953	0.801
		BA02	0.930			
		BA03	0.944			
		BA04	0.934			
		BA05	0.938			
2B	Intimacy	INT01	0.910	0.911	0.944	0.848
		INT02	0.928			
		INT03	0.925			
	Trust	TRU01	0.932	0.904	0.940	0.838
		TRU02	0.910			
		TRU03	0.904			
	Brand Attitude	BA01	0.913	0.961	0.970	0.865
		BA02	0.963			
		BA03	0.944			
		BA04	0.900			
		BA05	0.930			
2C	Intimacy	INT01	0.970	0.959	0.974	0.925
		INT02	0.946			
		INT03	0.969			
	Trust	TRU01	0.970	0.949	0.967	0.908
		TRU02	0.947			
		TRU03	0.942			
	Brand Attitude	BA01	0.955	0.969	0.976	0.891
		BA02	0.957			
		BA03	0.940			
		BA04	0.951			
		BA05	0.916			
2D	Intimacy	INT01	0.956	0.925	0.952	0.869
		INT02	0.907			
		INT03	0.934			
	Trust	TRU01	0.949	0.953	0.969	0.914
		TRU02	0.968			
		TRU03	0.951			
	Brand Attitude	BA01	0.941	0.963	0.971	0.870
		BA02	0.934			
		BA03	0.91			
		BA04	0.955			
		BA05	0.922			

2A: Eye Movement applied and Conversational Distance close. 2B: Eye Movement unapplied and Conversational Distance far. 2C: Eye Movement unapplied and Conversational Distance close. 2D: Eye Movement applied and Conversational Distance far.

**Table A4:** Discriminant Validity of Full Sample (N = 45).

Construct	HTMT
Intimacy → Brand Attitude	0.659
Trust → Brand Attitude	0.763
Trust → Intimacy	0.402

**Table A5:** Discriminant Validity of each case in Delivery Service Scenario.

Case	Construct	HTMT
1A	Intimacy → Brand Attitude	0.781
	Trust → Brand Attitude	0.688
	Trust → Intimacy	0.406
1B	Intimacy → Brand Attitude	0.578
	Trust → Brand Attitude	0.802
	Trust → Intimacy	0.308
1C	Intimacy → Brand Attitude	0.663
	Trust → Brand Attitude	0.763
	Trust → Intimacy	0.482
1D	Intimacy → Brand Attitude	0.711
	Trust → Brand Attitude	0.862
	Trust → Intimacy	0.607

1A: Eye Movement applied and Conversational Distance close. 1B: Eye Movement unapplied and Conversational Distance far. 1C: Eye Movement unapplied and Conversational Distance close. 1D: Eye Movement applied and Conversational Distance far.

**Table A6:** Discriminant Validity of each case in Public Transportation Service Scenario.

Case	Construct	HTMT
2A	Intimacy → Brand Attitude	0.617
	Trust → Brand Attitude	0.704
	Trust → Intimacy	0.316
2B	Intimacy → Brand Attitude	0.565
	Trust → Brand Attitude	0.794
	Trust → Intimacy	0.459
2C	Intimacy → Brand Attitude	0.684
	Trust → Brand Attitude	0.689
	Trust → Intimacy	0.259
2D	Intimacy → Brand Attitude	0.629
	Trust → Brand Attitude	0.809
	Trust → Intimacy	0.386

2A: Eye Movement applied and Conversational Distance close. 2B: Eye Movement unapplied and Conversational Distance far. 2C: Eye Movement unapplied and Conversational Distance close. 2D: Eye Movement applied and Conversational Distance far.

**Table A7:** Predictive Relevance of Full Sample (N = 45).

Construct	Q^2^
Brand Attitude	0.557
Trust	0.114

**Table A8:** Predictive Relevance of each case in Delivery Service Scenario.

Case	Construct	Q^2^
1A	Brand Attitude	0.518
	Trust	0.07
1B	Brand Attitude	0.543
	Trust	0.051
1C	Brand Attitude	0.551
	Trust	0.155
1D	Brand Attitude	0.622
	Trust	0.269

1A: Eye Movement applied and Conversational Distance close. 1B: Eye Movement unapplied and Conversational Distance far. 1C: Eye Movement unapplied and Conversational Distance close. 1D: Eye Movement applied and Conversational Distance far.

**Table A9:** Predictive Relevance of each case in Public Transport Service Scenario.

Case	Construct	Q^2^
2A	Brand Attitude	0.437
	Trust	0.041
2B	Brand Attitude	0.501
	Trust	0.135
2C	Brand Attitude	0.596
	Trust	0.038
2D	Brand Attitude	0.564
	Trust	0.106

2A: Eye Movement applied and Conversational Distance close. 2B: Eye Movement unapplied and Conversational Distance far. 2C: Eye Movement unapplied and Conversational Distance close. 2D: Eye Movement applied and Conversational Distance far.

**Table A10:** Tests of Within-Subjects Effects for the Full Sample (N = 45).

Source	Measures	F Statistic F(1, 44)	Significance Level (*p*-Value)	Partial Eta Square (η_p_^2^)
SCENE	INT	5.191	0.028 *	0.106
	TRU	1.639	0.207	0.036
	BA	0.618	0.436	0.014
EyeM	INT	7.707	0.008 **	0.149
	TRU	1.845	0.181	0.040
	BA	3.548	0.066	0.075
ConvD	INT	0.109	0.742	0.002
	TRU	0.143	0.707	0.003
	BA	0.003	0.959	0.000
SCENE * EyeM	INT	1.894	0.176	0.041
	TRU	0.073	0.788	0.002
	BA	1.070	0.307	0.024
SCENE * ConvD	INT	0.340	0.563	0.008
	TRU	3.317	0.075	0.070
	BA	0.498	0.484	0.011
EyeM * ConvD	INT	27.991	0.000 ***	0.389
	TRU	23.121	0.000 ***	0.344
	BA	17.529	0.000 ***	0.285
SCENE * EyeM * ConvD	INT	0.149	0.702	0.003
	TRU	0.010	0.919	0.000
	BA	0.013	0.909	0.000

SCENE: Scenario, EyeM: Eye Movement, ConvD: Conversational Distance. INT: Intimacy, TRU: Trust, BA: Brand Attitude. * *p* < 0.05, ** *p* < 0.01, *** *p* < 0.001.

**Table A11:** Tests of Within-Subjects Effects for Pick-up service Scenario (N = 45).

Source	Measures	F Statistic F(1, 44)	Significance Level (*p*-Value)	Partial Eta Square (η_p_^2^)	Observed Power
EyeM	INT	3.835	0.057	0.080	0.793
	TRU	1.545	0.220	0.034	0.105
	BA	1.573	0.216	0.035	0.481
ConvD	INT	0.007	0.932	0.000	0.085
	TRU	0.536	0.468	0.012	0.225
	BA	0.162	0.689	0.004	0.063
EyeM * ConvD	INT	11.890	0.001 **	0.213	0.989
	TRU	14.934	0.000 ***	0.253	0.837
	BA	9.718	0.003 **	0.181	0.850

EyeM: Eye Movement, ConvD: Conversational Distance. INT: Intimacy, TRU: Trust, BA: Brand Attitude. * *p* < 0.05, ** *p* < 0.01, *** *p* < 0.001.

**Table A12:** Tests of Within-Subjects Effects for Public Shuttle Scenario (N = 45).

Source	Measures	F Statistic F(1, 44)	Significance Level (*p*-Value)	Partial Eta Square (η_p_^2^)	Observed Power
EyeM	INT	8.607	0.007 **	0.155	0.482
	TRU	0.492	0.487	0.011	0.229
	BA	3.821	0.057	0.080	0.233
ConvD	INT	0.309	0.581	0.007	0.051
	TRU	1.509	0.226	0.033	0.111
	BA	0.119	0.732	0.003	0.068
EyeM * ConvD	INT	18.963	0.00 ***	0.301	0.921
	TRU	9.063	0.004 **	0.171	0.966
	BA	9.389	0.004 **	0.176	0.862

EyeM: Eye Movement, ConvD: Conversational Distance. INT: Intimacy, TRU: Trust, BA: Brand Attitude. * *p* < 0.05, ** *p* < 0.01, *** *p* < 0.001.
